# Rickettsioses in South Korea, Data Analysis

**DOI:** 10.3201/eid1203.050957

**Published:** 2006-03

**Authors:** Jui-Shan Ma

**Affiliations:** * Show-Chwan Memorial Hospital, Changhua, Taiwan

**Keywords:** SFG rickettsioses, Korea, rompB gene

**To the Editor:** Choi et al. (*1*) conducted a study on sequence analysis of a partial *rompB* gene amplified from sera of humans who were seropositive for spotted fever group (SFG) and typhus group rickettsioses. They write, "These finding suggested that several kinds of rickettsial diseases, including boutonneuse fever, rickettsialpox, *R. felis* infection, and Japanese spotted fever… are occurring in Korea."

These claims propagate some errors and may lead to an inadequate conclusion. First, *rompB* is conserved in *Rickettsia* spp. and consists of 4,968 bp with respect to the published sequence of the *R. conorii* strain Seven (*2**,**3*). Fournier et al. (*4*) amplified 4,682 bp of *rompB* and showed a high degree of nucleotide sequence similarity (99.2%) between *R. africae* and *R. sibirica*, *R. pakeri*, and *R. slovaca*. Choi et al. amplified ≈420 bp of *rompB* (position 3562–4077) for sequence analysis. This segment is located in a highly conserved region of the gene, which may decrease the ability to differentiate particular species from other SFG rickettsiae. This study cannot prove the existence of specific SFG rickettsioses until the results are confirmed further by, for example, isolating these SFG rickettsiae from humans, animals, or ticks in South Korea. Recently, the authors analyzed nucleotide sequences of 267-bp amplicons of *rompB* (position 4762–4496) obtained from patient sera and found that *R. conorii* could not be differentiated from *R. sibirica* (*5*). This finding also supports our concerns.

Second, although partial *rompB* nucleotide sequence analysis of rickettsiae obtained from 1 patient's serum showed 98.87% similarity with *R. conorii* strain Seven, the finding does not indicate boutonneuse fever is occurring in South Korea because high similarities (98.6%–99.8%) are found among 4 subspecies of *R. conorii*. Multilocus sequence typing can help differentiate among these subspecies (*6*).

This study provided a model to amplify SFG rickettsial DNA from sera of patients, and it will be helpful in surveillance of these diseases. However, the results should be interpreted more carefully in the context of clinical and epidemiologic data and combined with different gene sequence analyses to obtain a reliable and specific diagnosis.
